# Healthcare costs and utilization associated with high-risk prescription opioid use: a retrospective cohort study

**DOI:** 10.1186/s12916-018-1058-y

**Published:** 2018-05-16

**Authors:** Hsien-Yen Chang, Hadi Kharrazi, Dave Bodycombe, Jonathan P. Weiner, G. Caleb Alexander

**Affiliations:** 1 Department of Health Policy & Management, Johns Hopkins Bloomberg School of Public Health, Baltimore, MD USA; 2 Center for Drug Safety and Effectiveness, Johns Hopkins University, Baltimore, MD USA; 3 Center for Population Health Information Technology, Johns Hopkins University, Baltimore, MD USA; 4 Department of Epidemiology, Johns Hopkins Bloomberg School of Public Health, 615 N. Wolfe Street W6035, Baltimore, MD 21205 USA; 5 Division of General Internal Medicine, Department of Medicine, Johns Hopkins Medicine, Baltimore, MD USA

**Keywords:** Chronic high-dose opioid users, Concomitant users of opioid and benzodiazepine, Opioid shoppers, Healthcare costs, Resource utilization

## Abstract

**Background:**

Previous studies on high-risk opioid use have only focused on patients diagnosed with an opioid disorder. This study evaluates the impact of various high-risk prescription opioid use groups on healthcare costs and utilization.

**Methods:**

This is a retrospective cohort study using QuintilesIMS health plan claims with independent variables from 2012 and outcomes from 2013. We included a population-based sample of 191,405 non-elderly adults with known sex, one or more opioid prescriptions, and continuous enrollment in 2012 and 2013. Three high-risk opioid use groups were identified in 2012 as (1) persons with 100+ morphine milligram equivalents per day for 90+ consecutive days (chronic users); (2) persons with 30+ days of concomitant opioid and benzodiazepine use (concomitant users); and (3) individuals diagnosed with an opioid use disorder. The length of time that a person had been characterized as a high-risk user was measured. Three healthcare costs (total, medical, and pharmacy costs) and four binary utilization indicators (the top 5% total cost users, the top 5% pharmacy cost users, any hospitalization, and any emergency department visit) derived from 2013 were outcomes. We applied a generalized linear model (GLM) with a log-link function and gamma distribution for costs while logistic regression was employed for utilization indicators. We also adopted propensity score weighting to control for the baseline differences between high-risk and non-high-risk opioid users.

**Results:**

Of individuals with one or more opioid prescription, 1.45% were chronic users, 4.81% were concomitant users, and 0.94% were diagnosed as having an opioid use disorder. After adjustment and propensity score weighting, chronic users had statistically significant higher prospective total (40%), medical (3%), and pharmacy (172%) costs. The increases in total, medical, and pharmacy costs associated with concomitant users were 13%, 7%, and 41%, and 28%, 21% and 63% for users with a diagnosed opioid use disorder. Both total and pharmacy costs increased with the length of time characterized as high-risk users, with the increase being statistically significant. Only concomitant users were associated with a higher odds of hospitalization or emergency department use.

**Conclusions:**

Individuals with high-risk prescription opioid use have significantly higher healthcare costs and utilization than their counterparts, especially those with chronic high-dose opioid use.

## Background

Over the last two decades, adverse events from prescription opioids have soared in the United States [1–3]. In 2015, more Americans died of these products than ever before [4]; in addition, roughly 3.8 million people misused pain relievers every month and more than 2 million individuals are estimated to have an opioid use disorder every year, the majority of whom are not yet in treatment [5]. Given the morbidity and mortality from prescription opioids, several studies have investigated the direct and indirect societal costs of prescription opioid use disorders. For example, two widely cited studies, using claims data and publicly available secondary sources, estimated that the societal costs of prescription opioid use disorders skyrocketed from $8.6 billion in 2001 to $56 billion in 2007, while healthcare costs increased from $2.6 billion to $25 billion [6, 7]. Further, one review estimated that individuals with an opioid use disorder had annual healthcare costs that were $14,054–$20,546 greater than their counterparts among the privately insured, while commensurate increases among those with Medicaid ranged from $5870 to $15,183 per year [8]. More recent studies suggest similar additional expenses associated with opioid use disorders, ranging from $10,627 [9] to $20,760 [10] per year. This problem is not unique to the United States; for example, across the five largest European countries, the estimates of the incremental healthcare costs associated with prescription opioid abuse ranged from €900 to €2551 per person per year [11]. However, despite insights from prior studies, many analyses have adopted diagnosis codes rather than prescription drug utilization to identify high-risk opioid users [9, 10, 12–17], yet the vast majority of individuals with opioid use disorders are yet to be diagnosed. In addition, most studies have not explored the association between the extent of an individuals’ high-risk use and their healthcare costs and resource utilization [9, 18–20]. Finally, in many analyses, comparisons between high-risk users and their counterparts have not been well controlled, increasing the likelihood of confounding [18, 19, 21].

In this study, we used longitudinal claims to characterize the healthcare costs and utilization among three groups of individuals with alternative measures of high-risk prescription opioid use. In addition to examining the overall associations, we were also interested in examining whether there was a dose–response relationship between the associations of interest.

## Methods

### Data

We used QuintilesIMS patient-level administrative claims, which are derived from participating health plans across the United States, including commercial plans and those contracting with Medicare and Medicaid; however, the fee-for-service portion of the Medicare or Medicaid data was not present in the QuintilesIMS data. We included enrollees from the largest multi-region commercial plan. Other than demographic and enrollment information, the database also included diagnosis, procedure, medication, and cost information from inpatient, outpatient, and Emergency Department (ED) settings. Patient information was de-identified to comply with the Health Insurance Portability and Accountability Act.

### Study design and subjects

This is a 2-year retrospective cohort study (2012–2013). We used 2012 data to construct baseline covariates and three indicators of high-risk opioid users; for each type of high-risk opioid use, we also assigned an individual to a four-level indicator representing the magnitude of their opioid use. We used 2013 data to define our outcomes. Among 1,267,605 enrollees with continuous medical and pharmacy enrollment in 2012 and 2013, we restricted to 893,835 (70.51%) individuals between 18 and 64 years of age and with known sex; among them, 191,405 (21.41%) enrollees had at least one prescription opioid claim in 2012.

### Identification of high-risk opioid users

High-risk group membership was identified using 2012 claims. First, we used files provided by the Centers for Disease Control and Prevention to define the prescription opioids and benzodiazepines of interest [22]. These files contained information on strength per unit and a morphine milligram equivalent (MME) conversion factor at the National Drug Code (NDC) level. The MME provides a standardized measure across quantities and strength. Next, we used the 2012 medical and pharmacy claims to define three patient groups at elevated risks of adverse events from prescription opioids. We defined ‘chronic users’ as those consuming more than 100 MMEs per day for more than 90 consecutive days [23, 24]. Chronic opioid use is related to higher medical utilization and a greater likelihood of overdose death [20, 21, 25]. We defined ‘concomitant users’ as patients filling more than 30-days of concomitant opioids and benzodiazepines [23, 24]. Benzodiazepines are associated with approximately one-third of overdose deaths involving prescription opioids [26] and the odds of dying by overdose is four-fold higher among veterans with current benzodiazepine prescriptions [27]. Finally, we defined ‘opioid disorders’ as patients with one or more diagnosis codes representing opioid use disorders (Appendix 1) [9, 12, 14–16, 28, 29].

We used two approaches to examine a dose–response association between high-risk opioid use and healthcare utilization. First, we categorized high-risk users into tertiles based on their magnitude of high-risk opioid use (level 1–3); we included non-high-risk opioid users as a reference group (level 0). For ‘chronic users’, we determined the number of days an individual was considered a chronic user to define the magnitude of high-risk opioid use; for ‘concomitant users’, we used the number of days with both drugs on hand; for those with opioid disorders, we adopted the number of months diagnosed with opioid use disorders. Second, we counted the number of high-risk groups each enrollee belonged to (0–3).

### Outcome variables

Outcomes were derived from 2013 claims data. We calculated total, medical, and pharmacy costs (we also calculated pharmacy costs associated with prescription opioids only) and four binary measures of utilization (being among the top 5% total cost users, being among the top 5% pharmacy cost users, having any hospitalization, and having any ED visit) as our outcome variables. The annual costs were derived from claims and represents the sum of allowed amounts, reflecting what the insurance plan paid for services.

### Control variables

We derived control variables from the Johns Hopkins Adjusted Clinical Group (ACG v11.0) Risk Adjustment System, using both medical and pharmacy claims as inputs. The ACG system is a widely used morbidity measure [30–37]; it has been validated against costs [33, 35, 36], utilization [32, 38], and death [39]. The ACG system assigns all ICD codes to one of 32 Aggregated Diagnostic Clusters (ADGs). Each ADG is a morbidity group consisting of clinically homogeneous diagnosis codes with a similar expected need for medical resources. Based on their ADGs, age, and sex, individuals are assigned to one of 93 discrete ACG categories. The ACG system also assigns each NDC code to one of 67 Rx-defined morbidity groups (RxMG) based on the combination of active ingredient and route of administration [30, 40]. The ACG system also calculates the number of chronic conditions/active ingredients using the diagnosis codes/NDC an individual encountered. In addition, the ACG system generates a risk score for concurrent total costs, including independent variables such as diagnosis-based overall disease burden, high-impact chronic conditions, diagnoses representing a high likelihood of hospitalization, and acute conditions.

### Statistical analysis

We first described the characteristics of all enrollees, enrollees with any opioid use, and high-risk opioid users. Then, we constructed statistical models to evaluate the impact of being a high-risk user and the magnitude of high-risk opioid use on the prospective healthcare costs and utilization.

We used propensity score weighting to control for baseline differences in patient characteristics between high-risk and non-high-risk opioid users. Logistic regression was applied to derive a propensity score of becoming a high-risk user based on patient sex, four age categories (18–34, 35–44, 45–54, and 55–64), 32 diagnosis-based ADGs, 67 medication-based RxMGs, number of chronic conditions, number of active ingredients, and a concurrent risk score. An individual had three propensity score weights corresponding to each measure of high-risk use. We chose propensity score weighting because we could include all high-risk users, as might not occur with matching, and we wanted one interpretable overall effect, which might be impossible with stratification. We derived an average treatment effect of the treated weighting because we could estimate the average effect of treatment on the treated subjects, thus making comparisons between the actual outcomes of high-risk users and the expected outcomes under the counterfactual if they had not been high-risk users. This is especially useful when the study sample differs systematically from the overall population [41].

We examined the performance of our propensity score weighting based on comparisons of (1) high-risk users versus non-high-risk users with an opioid prescription and (2) high-risk users versus enrollees without an opioid prescription. We restricted our analyses to the first comparison because, after applying propensity score weighting, the average of the absolute standardized differences of all variables in the models reduced to less than 0.1; such differences were larger for the second comparison.

We used a generalized linear model (GLM) with a log-link function and gamma distribution to model costs, given the non-negative and positively skewed distribution of costs as well as a much higher proportion of people with very high costs [42–44]. We added $1 to all costs so that non-users could be included in the model. The log-link function provides an estimate of the proportional change in mean costs. We applied logistic regression for binary utilization indicators and included the same set of covariates from the propensity score model to control for residual confounding. We constructed both a crude and adjusted model with covariates and weights to explore the relationship between being high-risk opioid users and each outcome. For analyses of the dose–response association, we constructed a crude mode and an adjusted model with covariates since propensity scores were usually generated for binary outcomes.

As a sensitivity analysis, we also performed multivariate linear regression to test the robustness of our findings, since it is widely used to analyze costs, delivers intuitive results, and often performs similarly as other statistical models (such as a two-part lognormal model) with a sufficiently large sample size [8, 9, 33, 45]. These results yielded substantive similar findings and are reported in Appendix 2, but not discussed further herein.

## Results

### Characteristics of the eligible participants

Of 893,835 eligible enrollees, we identified 0.31% chronic users, 1.03% concomitant users, and 0.27% individuals with a diagnosis of an opioid disorder (Table 1). Of the 191,405 enrollees with any opioid use, the respective proportions of individuals with chronic use (2778/1.45%), concomitant use (9200/4.81%), and an opioid use disorder (1798/0.94%) were greater. The mean age of eligible enrollees was 42.4 years old, and about half (49.52%) were female; enrollees with any opioid prescription were slightly older (44.2 years old) and mostly female (55.14%). Individuals with opioid disorders were more likely to be younger and male than those in the other two high-risk groups.

**Table 1:** Characteristics of three high-risk opioid groups, patients with any opioid and the whole study sample

	Chronic users	Concomitant users	Opioid disorders	Patients with any opioid use	All patients
Number of study subjects	2778	9200	1798	191,405	893,835
Age	47.22 (10.60)	49.47 (9.64)	38.59 (12.90)	44.16 (12.64)	42.43 (13.09)
Female	47.16%	65.76%	42.83%	55.14%	49.52%
*Concurrent medical utilization in 2012*					
Total cost	30,486 (57,670)	28,818 (61,406)	29,097 (50,380)	14,005 (34,238)	5456 (19,116)
Medical cost	19,275 (53,908)	22,195 (57,710)	23,662 (49,056)	11,888 (32,241)	4321 (17,632)
Pharmacy cost	11,211 (15,483)	6623 (15,638)	5435 (8119)	2117 (8120)	1135 (5357)
Opioid medication cost	6169 (11,315)	1393 (5560)	2494 (4158)	169 (1622)	36 (754)
1+ Hospitalization	15.95%	17.59%	29.14%	9.28%	2.98%
1+ Emergency visit	29.84%	33.55%	40.71%	27.76%	12.36%
*Prospective medical utilization in 2013*					
Total cost	31,045 (69,822)	27,040 (58,601)	26,061 (61,520)	11,176 (37,069)	5972 (23,001)
Medical cost	19,663 (65808)	20,008 (54,226)	20,758 (60,462)	8896 (34,402)	4739 (21,325)
Pharmacy cost	11,382 (17,539)	7033 (18,156)	5302 (7968)	2280 (10,617)	1233 (6530)
Opioid medication cost	6079 (13,170)	1459 (6506)	2416 (4606)	174 (1842)	40 (857)
1+ Hospitalization	14.25%	15.03%	17.96%	6.10%	3.03%
1+ Emergency visit	27.54%	31.07%	34.32%	18.61%	12.01%
*Concurrent morbidity in 2012*					
Count of ADGs	8.40 (4.34)	9.40 (4.22)	8.73 (4.36)	6.55 (3.78)	4.12 (3.49)
Count of RxMGs	8.85 (4.53)	10.31 (4.13)	7.77 (4.51)	5.98 (3.41)	3.00 (3.09)
Count of chronic conditions	4.00 (3.11)	4.43 (3.13)	4.11 (2.95)	2.26 (2.40)	1.31 (1.85)
Count of active ingredients	13.10 (8.16)	15.84 (8.09)	11.65 (8.37)	8.42 (5.79)	4.03 (4.64)
Concurrent risk score	4.67 (6.81)	4.95 (6.96)	5.16 (6.77)	2.49 (4.33)	1.14 (2.66)
*Prospective morbidity in 2013*					
Count of ADGs	8.25 (4.52)	9.13 (4.45)	7.73 (4.63)	5.86 (4.01)	4.18 (3.58)
Count of RxMGs	8.76 (4.64)	10.02 (4.41)	7.15 (4.65)	5.07 (3.92)	3.12 (3.19)
Count of chronic conditions	4.03 (3.26)	4.45 (3.26)	3.64 (3.11)	2.20 (2.48)	1.41 (1.96)
Count of active ingredients	12.75 (8.29)	15.17 (8.38)	10.42 (8.12)	7.13 (6.34)	4.18 (4.81)
Concurrent risk score	4.75 (7.42)	4.88 (7.10)	3.99 (6.22)	2.17 (4.33)	1.22 (2.95)
*Overlap between three high-risk opioid groups in 2012*					
Chronic users	–	13.33%	29.48%	1.45%	0.31%
Concomitant users	44.13%	–	25.80%	4.81%	1.03%
Opioid disorders	19.08%	5.04%	–	0.94%	0.27%
*Magnitude of high-risk use in 2012*	115.72 (85.23) / Days	150.72 (102.72) / Days	3.74 (3.29) / Months	–	–
*Count of high-risk user membership in 2012*					
Count of membership	1.63 (0.60)	1.18 (0.43)	1.55 (0.66)	0.07 (0.30)	0.02 (0.14)
0	–	–	–	93.87%	98.62%
1	43.05%	83.52%	54.39%	5.15%	1.17%
2	50.68%	14.59%	35.93%	0.89%	0.19%
3	6.26%	1.89%	9.68%	0.09%	0.02%

Of the three high-risk groups examined, individuals with opioid use disorders had the lowest total ($25,000+) and pharmacy (~$4400) costs concurrently and prospectively; chronic users had the highest total (~$30,000) and pharmacy costs (~$11,000), but the lowest medical costs (~$20,000). Notably, more than one-third of chronic users’ total costs were due to pharmacy costs, while pharmacy costs of those with opioid use disorders accounted for less than one-fifth of their total costs. In addition, the amount of pharmacy costs accounted for by prescription opioids varied across the three groups, with chronic users having the greatest proportion (more than 50%), compared to individuals with opioid use disorders (45%) and concomitant users (~20%).

Chronic users had lower rates of concurrent and prospective hospital (~15%) and ED (~28%) utilization, while those with opioid disorders had the highest rates of such utilization, although they declined over time. For example, among those with opioid disorders, the rates of hospitalization decreased from 29% concurrently to 18% prospectively, and the rates of ED visits decreased from 41% concurrently to 34% prospectively. Among the three high-risk groups, concomitant users had the highest morbidity across five ACG-based indicators, both concurrently and prospectively, while patients with opioid use disorders had the lowest morbidity prospectively.

The overlap across these three high-risk groups was low; the highest was 44.13% for chronic users being also concomitant users while the lowest was 5.04% for concomitant users being patients with opioid disorders. In the concurrent year, chronic users had 116 days being considered as chronic users, concomitant users had 150 days being concomitant users, and patients with opioid disorders had 3.7 months with a diagnosis of opioid disorders. On average, chronic users were included in 1.63 high-risk groups, concomitant users in 1.18, and patients with opioid disorders in 1.55.

### Prospective healthcare costs associated with high-risk use

High-risk opioid use was associated with statistically significant increases in prospective total, medical, and pharmacy costs (Table 2). For example, after adjustment and weighting, chronic use was associated with greater prospective total (40%), medical (3%), and pharmacy (172%) costs. Similarly, corresponding increased costs were 13% (total costs), 7% (medical costs), and 41% (pharmacy costs) for concomitant users, and 28% (total costs), 21% (medical costs), and 63% (pharmacy costs) for patients with opioid use disorders.

**Table 2:** Impact of being high-risk opioid users and its magnitude on prospective costs

	Total cost	Medical cost	Drug cost
*Crude cost ratio – binary indicator*			
Chronic users	2.85** (2.69–3.03)	2.25** (2.11–2.40)	5.30** (4.96–5.67)
Concomitant users	2.61** (2.52–2.69)	2.40** (2.32–2.49)	3.45** (3.32–3.58)
Opioid disorder	2.36** (2.19–2.54)	2.36** (2.18–2.56)	2.36** (2.17–2.56)
*Adjusted cost ratio (with covariates and weighting) – binary indicator*			
Chronic users	1.40** (1.39–1.42)	1.03* (1.02–1.04)	2.72** (2.69–2.75)
Concomitant users	1.13** (1.12–1.14)	1.07** (1.06–1.08)	1.41** (1.40–1.43)
Opioid disorder	1.28** (1.26–1.29)	1.21** (1.20–1.23)	1.63** (1.62–1.65)
*Crude cost ratio (one level/count increase in magnitude/membership: 0–3)*			
Magnitude of chronic users	1.62** (1.57–1.66)	1.44** (1.39–1.49)	2.17** (2.10–2.25)
Magnitude of concomitant users	1.54** (1.52–1.57)	1.48** (1.45–1.50)	1.76** (1.73–1.79)
Magnitude of opioid disorder	1.48** (1.43–1.54)	1.48** (1.42–1.54)	1.48** (1.42–1.54)
Count of high-risk group membership	2.18** (2.12–2.23)	2.02** (1.96–2.07)	2.81** (2.73–2.89)
*Adjusted cost ratio (with covariates; one level/count increase in magnitude/membership: 0–3)*			
Magnitude of chronic users	1.19** (1.16–1.23)	1.01 (0.98–1.04)	1.79** (1.74–1.84)
Magnitude of concomitant users	1.04** (1.03–1.06)	1.02 (1.00–1.03)	1.14** (1.13–1.16)
Magnitude of opioid disorder	1.19** (1.14–1.23)	1.20** (1.15–1.26)	1.27** (1.22–1.32)
Count of high-risk group membership	1.17** (1.14–1.20)	1.06** (1.03–1.10)	1.64** (1.59–1.68)

*p < 0.05; **p < 0.01

Total and pharmacy costs, but not medical costs, increased as the magnitude of high-risk opioid use increased. After adjustment, each additional level in magnitude showed a statistically significant association with 19%/79% increases in total/pharmacy costs among chronic users, 4%/14% increases among concomitant users, and 19%/27% increases among patients with opioid use disorders. Costs were also greater among individuals with multiple high-risk group membership; each additional membership was statistically significantly associated with a 17% increase in total costs, a 6% increase in medical costs, and a 64% increase in pharmacy costs.

### Prospective medical utilization associated with high-risk use

High-risk users were also associated with a statistically significantly greater likelihood of falling into the top fifth percentile of total and pharmacy spending, but only concomitant users had a statistically significantly higher likelihood of having any hospitalization and any emergency room visit (Table 3). For example, after accounting for covariates and weights, concomitant users were statistically significantly more likely to fall into the top 5% of total spending (odds ratio (OR) 1.18, 95% confidence interval (CI) 1.08–1.29) and pharmacy spending (OR 1.61, CIs 1.48–1.75), use an inpatient service (OR 1.09, CIs 1.00–1.19), or visit the ED (OR 1.13, CI 1.06–1.21). Similar patterns were observed when examining the association between the magnitude of high-risk use and medical utilization; individuals with greater high-risk use or membership in various high-risk groups were statistically significantly more likely to fall into the top fifth percentile of total and pharmacy costs, but not necessarily have greater odds of any hospitalization or ED utilization.

**Table 3:** Impact of being high-risk opioid users and its magnitude on prospective medical utilization

	Top 5% total cost	Top 5% drug cost	Any hospitalization	Any emergency visit
*Crude odds ratio (without covariates) – binary indicator*				
Chronic users	4.07** (3.68–4.50)	13.58** (12.55–14.70)	2.61** (2.35–2.91)	1.68** (1.54–1.82)
Concomitant users	3.92** (3.69–4.16)	5.97** (5.65–6.31)	2.96** (2.78–3.14)	2.06** (1.96–2.15)
Opioid disorder	2.92** (2.54–3.35)	3.63** (3.19–4.13)	3.44** (3.04–3.88)	2.31** (2.09–2.55)
*Adjusted odds ratio (with covariates and weighting) – binary indicator*				
Chronic users	1.41** (1.20–1.66)	4.97** (4.31–5.74)	1.03 (0.87–1.20)	1.00 (0.89–1.14)
Concomitant users	1.18** (1.08–1.29)	1.61** (1.48–1.75)	1.09* (1.00–1.19)	1.13** (1.06–1.21)
Opioid disorder	1.20 (0.95–1.50)	1.65** (1.32–2.06)	0.94 (0.78–1.13)	1.03 (0.88–1.21)
*Crude odds ratio (one level/count increase in magnitude/membership: 0–3)*				
Magnitude of chronic users	1.79** (1.71–1.87)	3.07** (2.96–3.19)	1.47** (1.40–1.54)	1.24** (1.19–1.29)
Magnitude of concomitant users	1.76** (1.72–1.81)	2.15** (2.10–2.20)	1.57** (1.53–1.61)	1.36** (1.33–1.39)
Magnitude of opioid disorder	1.52** (1.43–1.62)	1.71** (1.61–1.81)	1.65** (1.56–1.74)	1.37** (1.31–1.44)
Count of high-risk group membership	2.62** (2.52–2.73)	4.14** (3.98–4.30)	2.20** (2.11–2.29)	1.69** (1.64–1.75)
*Adjusted odds ratio (with covariates; one level/count increase in magnitude/membership: 0–3)*				
Magnitude of chronic users	1.18** (1.11–1.25)	2.53** (2.41–2.67)	0.97 (0.92–1.03)	1.01 (0.97–1.06)
Magnitude of concomitant users	1.06** (1.03–1.10)	1.27** (1.23–1.31)	1.00 (0.97–1.04)	1.05** (1.03–1.08)
Magnitude of opioid disorder	1.12* (1.02–1.24)	1.27** (1.16–1.39)	1.11* (1.02–1.10)	0.99 (0.93–1.06)
Count of high-risk group membership	1.17** (1.10–1.25)	2.22** (2.09–2.35)	1.02 (0.96–1.08)	1.08** (1.03–1.13)

*p < 0.05; **p < 0.01

## Discussion

We used a large, commercially insured population to identify and characterize three groups of high-risk opioid users: ‘chronic high-dose users’, ‘concomitant users’ (of opioids and benzodiazepines), and individuals diagnosed with an opioid use disorder. All high-risk use was associated with much higher prospective pharmacy and total costs and, among the three groups examined, costs were greatest among chronic users (40% increase in total costs) and modestly lower among those with a diagnosed opioid use disorder (28%) or concomitant users (13%). In addition, high-risk use was associated with a greater likelihood of falling into the top fifth percentile of spending but not necessarily having higher resource use. Similar patterns were observed between a longer duration of such use and a greater likelihood of higher healthcare costs and resource utilization. These results are important given how commonly high-risk use occurs and the significant questions that remain regarding its impact on healthcare costs and utilization.

Most of the available studies identifying patients with opioid disorders rely on similar diagnosis codes. However, the definition of chronic users varies as do their denominators; for example, chronic users were defined as having 180+ days of supply among the insured with a medical or pharmacy claim [21], 100 MMEs or above per day in a year among enrollees with an opioid prescription [20], or at least 120 days of opioid prescription over any continuous 6 month period among enrollees with continuous enrollment [46]. Thus, the prevalence of patients diagnosed with opioid disorders in our study was on par with those from previous studies, ranging from 0.16% to 0.87% [9, 10, 16, 47], while that of chronic users was considerably different to those reported (0.65–2.8%) [20, 21, 46]; these differences must be taken into account when making comparisons across studies.

Out of 2378 eligible enrollees with a diagnosis of opioid disorders, 580 (24.39%) did not have any opioid prescription; thus, they were excluded from our analysis and the final sample size for patients with opioid disorders was reduced to 1798. This phenomenon has been reported by other researchers; indeed, in one study, approximately one-third of patients with an opioid abuse diagnosis did not have any opioid prescription [16], while in another study, 0.01% of the pharmacy-based non-opioid group still had a diagnosis of opioid abuse [21]. Among the 580 patients that did not have any opioid prescription, about two-fifths did not have their first diagnosis until July 2012 or later; therefore, these patients had enough time to obtain an opioid prescription prior to the first diagnosis, assuming that opioids would not be prescribed for patients with such diagnoses. It is likely that the illicit use of prescription opioid plays an important role in this finding, as approximately 3.8 million people misused pain relievers every month in 2015 [5].

Our findings extend previous analyses of the direct costs of prescription opioid use, although we used both prescription and medical claims to define our groups and adopted a more comprehensive set of covariates to decrease the potential for confounding. Based on our results, we estimated that the incremental total costs among patients with chronic use, concomitant use, and an opioid use disorder were $8870, $3111, and $5385, respectively. Our estimates are lower than prior estimates, which have generally ranged from $10,627 to $20,546 [9, 10, 16, 46]; such reductions may be in part due to better adjustment for potential confounders and our selection of a comparison group. For example, many prior studies have matched groups based on demographics only [10, 16, 17, 20], while even those matching on morbidity or baseline utilization [9] have only reported simple differences in average costs between high- and low-risk groups, leading to possible residual confounding [9, 10, 16, 20, 46]. In addition, others have used comparison groups including enrollees with an opioid claim [10, 17, 20], any medical claim [9], any medical or pharmacy claim [21], or just continuous enrollment [46]; however, these studies have not evaluated the comparability of their low- and high-risk groups. On the other hand, our estimate that individuals with an opioid use disorder had 28% greater total costs is similar to what was reported in another study with matching and outcome modelling [17].

Healthcare payers are an increasingly important stakeholder in the opioid epidemic, given the financial incentives to manage the resource use associated with the epidemic and the imperative to reduce injuries and deaths from these products. Health plans have access to patients’ healthcare and prescription drug utilization, which allows for observation of events such as ED visits or hospitalizations associated with an opioid injury, as well as the quality and comprehensiveness of their primary care. Payers can engage to improve the care of high-risk patients, including minimizing early opioid exposure, using surveillance to identify high-risk prescribing and utilization, and providing greater assistance to those with opioid use disorders. For example, ‘First-fill’ education programs and early interventions to decrease patient’s progression from acute to chronic use offer an important window of opportunity given the risks of long-term use associated with early fill patterns [48, 49]. Provider-targeted programs are also important since provider prescribing is associated with the patients’ long-term use [50], and the remarkable variation in opioid prescribing across providers [51–53]. Warm hand-offs [54] and early access to comprehensive medication assisted treatment after overdose [55] have also been promoted, given the high proportion of patients that resume prescription opioid use after an opioid-related adverse event [56] and the potential for patients to resume prescription opioid use during or following periods of buprenorphine receipt [57].

Our study had several limitations. First, we identified high-risk opioid use through claims data and thus could not identify the non-medical use of opioids diverted from friends or family. Our data also do not allow for us to examine heroin or illicit fentanyl use, and it is unclear to what degree we underestimate the true costs and resource use associated with the epidemic. Second, since we only had 2 years of data, we did not evaluate the long-term impact of high-risk opioid use. Third, we excluded Medicare and Medicaid enrollees and thus our results are not generalizable to all opioid users, but rather, reflect the experience of a group of commercially insured individuals; future work among the Medicare and Medicaid enrollees would provide important information. Fourth, studies have used different methods to define high-risk groups, yielding different estimates of their prevalence [9, 10, 16, 20, 21, 46, 47], making the direct comparison of estimates across studies difficult. Fifth, by requiring 2-year continuous enrollment, we excluded patients who died or lost job/insurance in 2013, which more likely concentrated on high-risk opioid users. Therefore, our results may underestimate the costs and utilization associated with high-risk opioid use. Sixth, even though we adopted propensity score weighting to control for baseline differences between groups, it is still possible that confounding exists, especially from those confounders not included in the propensity score model. Finally, we did not examine the experience of another high-risk opioid user group ‘opioid shoppers’, who obtain opioid prescriptions from multiple pharmacies and providers, since our data did not include a consistent means of identifying individual providers and pharmacies.

## Conclusions

Three measures of high-risk prescription opioid use are associated with greater prospective costs. While our findings were modestly lower than previous estimates, the total cost of high-risk users was 4–6 times higher than that of all eligible enrollees and average pharmacy costs were as much as 4- to 11-fold higher. Given that opioid abuse-related injuries and deaths show no signs of abating, our findings underscore yet another dimension of the epidemic and the value of payer-driven interventions to reverse it.

## Appendix 1

### ICD-9-CM codes used to identify patients with ‘opioid disorders’

Opioid-type dependence (304.0X).

Combination of opioid abuse with any other (304.7X).

Opioid abuse (305.5X).

Poisoning by opiates and related narcotics, not heroin (965.0, 965.00, 965.02, 965.09)

## Appendix 2

**Table 4:** Impact of being high-risk opioid group and its severity on prospective costs

	Total cost	Medical cost	Drug cost
*Crude cost change – binary indicator*			
Chronic users	20,161** (18,775–21,547)	10,925** (9637–12,213)	9236** (8841–9632)
Concomitant users	16,666** (15,893–17,439)	11673** (10,954–12,392)	4993** (4772–5214)
Opioid disorder	15,026** (13,306–16,746)	11,974** (10,377–13,571)	3051** (2559–3544)
*Adjusted cost change (with covariates and weighting) – binary indicator*			
Chronic users	6216** (5678–6754)	281 (−123 to 795)	5935** (5808–6063)
Concomitant users	637* (134–1140)	-1036** (−1518 to −553)	1673** (1546–1799)
Opioid disorder	1558** (1039–2076)	671** (161–1181)	887** (809–964)
*Crude cost change (one level increase in severity) – severity level (0–3)*			
Severity of chronic users	8845** (8205–9486)	4523** (3928–5119)	4322** (4139–4505)
Severity of concomitant users	7343** (6987–7700)	4959** (4628–5291)	2384** (2282–2486)
Severity of opioid disorder	6206** (5390–7022)	4880** (4123–5637)	1326** (1093–1560)
*Adjusted cost change (with covariates: one level increase in severity) – severity level (0–3)*			
Severity of chronic users	3337** (2695–3978)	263 (−344 to 869)	3074** (2887–3261)
Severity of concomitant users	1209** (840–1579)	315 (−34 to 664)	895** (787–1002)
Severity of opioid disorder	1136* (206–2067)	809 (−70 to 1688)	328* (56–599)

* *p* < 0.05; ** *p* < 0.01
